# Dr. Miles D. Goodyear

**Published:** 1914-02

**Authors:** 


Dr. Miles D. Goodyear, Michigan 1868, died November 25, 1913, at his home in Groton, aged 67.

